# (*E*)-3-(2,4-Dimeth­oxy­phen­yl)-1-(3,4-dimeth­oxy­phen­yl)prop-2-en-1-one

**DOI:** 10.1107/S1600536810043606

**Published:** 2010-10-31

**Authors:** Xiaokai Wu, Xiaoqing Cai, Xianan Zheng, Zhennan Zhang, Xiaoqin Ye

**Affiliations:** a School of Pharmacy, Wenzhou Medical College, Wenzhou, Zhejiang 325035, People’s Republic of China; b College of Chemistry and Materials Engineering, Wenzhou University, Wenzhou 325035, People’s Republic of China

## Abstract

The title compound, C_19_H_20_O_5_, is approximately planar; the dihedral angle between the benzene rings is 3.82 (8)°, and the central propenone C(=O)—C=C plane makes dihedral angles of 1.95 (10) and 3.17 (11)° with the two benzene rings. In the crystal structure, intra- and inter­molecular C—H⋯O hydrogen bonds are observed.

## Related literature

For related structures, see: Huang *et al.* (2010 ▶); Peng *et al.* (2010 ▶); Yathirajan *et al.* (2006 ▶); Zhao *et al.* (2010 ▶). For background to and applications of chalcones, see: Liang *et al.* (2007 ▶); Liu *et al.* (2008 ▶); Mojzisa *et al.* (2008 ▶); Nielsen *et al.* (2005 ▶); Nowakowska (2007 ▶); Selvakumar *et al.* (2007 ▶); Wu *et al.* (2010 ▶); Wu, Chen *et al.* (2009 ▶); Wu, Qiu *et al.* (2009 ▶); Wu, Zhang *et al.* (2009 ▶).
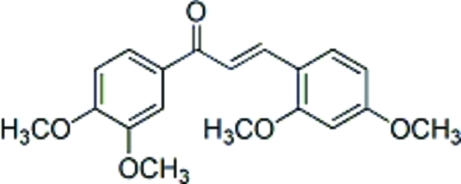


## Experimental

### 

#### Crystal data

C_19_H_20_O_5_
*M* *_r_* = 328.35
Monoclinic, 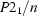

*a* = 9.031 (5) Å
*b* = 7.962 (5) Å
*c* = 23.631 (14) Å
β = 92.827 (10)°
*V* = 1697.0 (17) Å^3^
*Z* = 4
Mo *K*α radiation
μ = 0.09 mm^−1^
*T* = 298 K
0.47 × 0.35 × 0.31 mm

#### Data collection

Bruker APEX area-detector diffractometer
Absorption correction: multi-scan (*SADABS*; Bruker, 2002 ▶) *T*_min_ = 0.958, *T*_max_ = 0.972
8581 measured reflections
2981 independent reflections
2407 reflections with *I* > 2σ(*I*)
*R*_int_ = 0.021

#### Refinement

*R*[*F* ^2^ > 2σ(*F* ^2^)] = 0.042
*wR*(*F* ^2^) = 0.119
*S* = 1.04
2981 reflections
222 parameters
H-atom parameters constrained
Δρ_max_ = 0.14 e Å^−3^
Δρ_min_ = −0.16 e Å^−3^

### 

Data collection: *SMART* (Bruker, 2002 ▶); cell refinement: *SAINT* (Bruker, 2002 ▶); data reduction: *SAINT*; program(s) used to solve structure: *SHELXS97* (Sheldrick, 2008 ▶); program(s) used to refine structure: *SHELXL97* (Sheldrick, 2008 ▶); molecular graphics: *SHELXTL* (Sheldrick, 2008 ▶); software used to prepare material for publication: *SHELXL97*.

## Supplementary Material

Crystal structure: contains datablocks I, global. DOI: 10.1107/S1600536810043606/is2604sup1.cif

Structure factors: contains datablocks I. DOI: 10.1107/S1600536810043606/is2604Isup2.hkl

Additional supplementary materials:  crystallographic information; 3D view; checkCIF report

## Figures and Tables

**Table 1:** Hydrogen-bond geometry (Å, °)

*D*—H⋯*A*	*D*—H	H⋯*A*	*D*⋯*A*	*D*—H⋯*A*
C4—H4⋯O3^i^	0.93	2.46	3.363 (3)	162
C8—H8*B*⋯O4^ii^	0.96	2.60	3.537 (3)	166
C10—H10⋯O2	0.93	2.25	2.846 (3)	121
C19—H19*A*⋯O1^iii^	0.96	2.54	3.443 (3)	157

Symmetry codes: (i) 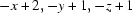; (ii) 
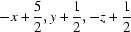
; (iii) 
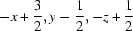
.

**Fig. 1.**
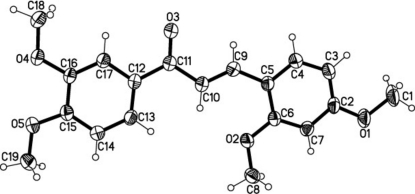
The molecular structure of the title compound, with displacement ellipsoids at the 50% probability level.

## supplementary crystallographic information

### Comment

Chalcones have the common skeleton of 1,3-diaryl-2-propen-1-ones and belong to the flavonoid family. Chalcones distribute widespread in fruits, vegetables and so on. Like as other flavonoids, chalcones have been reported to possess wide-range biological activities, including antimicrobial, antitumor, anti-inflammatory, antifungal, antioxidant activities and so on (Nowakowska, 2007; Liu *et al.*, 2008; Wu *et al.*, 2010). Moreover, Chalcones belong to nature products and have low toxicity. Owing to its varied pharmacological activities and low toxicity, it has attracted more and more scientists attention and therefore several strategies have been developed to synthesize them (Nowakowska, 2007; Selvakumar *et al.*, 2007; Wu, Chen *et al.*, 2009; Wu, Qiu *et al.*, 2009; Wu, Zhang *et al.*, 2009).

In our effort to develop Chalcones activity, we have synthesized the title chalcone. In order to get detailed information such as the geometrical features and the underlying interaction of the crystal structure, an X-ray study of the title compound was carried out.

Two rings of molecule is approximately planar and the dihedral angle between the two rings is 3.82 (4)°. The average value of exocyclic bond angles [120.8 (5)°] and the bond distances [1.384 (2) Å] in the phenyl rings agree well with the normal values reported in the literature for some analogous structures (Peng *et al.*, 2010; Wu, Chen *et al.*, 2009; Wu, Qiu *et al.*, 2009; Wu, Zhang *et al.*, 2009; Huang *et al.*, 2010; Yathirajan *et al.*, 2006).

### Experimental

The title compound was synthesized by Claisene–Schmidt condensation between 2,4-dimethoxybenzaldehyde and 1-(3,4-dimethoxyphenyl)ethanone. 2,4-Dimethoxybenzaldehyde (1 mmol) and 1-(3,4-dimethoxyphenyl)ethanone (1 mmol) were dissolved in ethanol (15 ml). The mixture were controlled at 279 K and then 5 drops NaOH (20%) was added. The reaction was monitored by thin-layer chromatography. 15 ml H_2_O was added after 10 h and the yellow solid precipitated was washed with water and cold ethanol. It was then dried and purified by column chromatography on silica gel. Single crystals of the title compound were grown in a CH_2_Cl_2_/CH_3_CH_2_OH mixture (1:1) solution at 279 K.

### Refinement

All H atoms were positioned geometrically and allowed to ride on their parent atoms at distances of 0.93 or 0.96 Å, with isotropic displacement parameters 1.2 or 1.5 times *U*_eq_ of the parent atom.

### Crystal data

**Table:**

C_19_H_20_O_5_	*F*(000) = 696
*M**_r_* = 328.35	*D*_x_ = 1.285 Mg m^−^^3^
Monoclinic, *P*2_1_/*n*	Mo *K*α radiation, λ = 0.71073 Å
Hall symbol: -P 2yn	Cell parameters from 3028 reflections
*a* = 9.031 (5) Å	θ = 2.4–25.4°
*b* = 7.962 (5) Å	µ = 0.09 mm^−^^1^
*c* = 23.631 (14) Å	*T* = 298 K
β = 92.827 (10)°	Block, colourless
*V* = 1697.0 (17) Å^3^	0.47 × 0.35 × 0.31 mm
*Z* = 4	

### Data collection

**Table:**

Bruker APEX area-detector diffractometer	2981 independent reflections
Radiation source: fine-focus sealed tube	2407 reflections with *I* > 2σ(*I*)
graphite	*R*_int_ = 0.021
φ and ω scans	θ_max_ = 25.0°, θ_min_ = 1.7°
Absorption correction: multi-scan (*SADABS*; Bruker, 2002)	*h* = −10→10
*T*_min_ = 0.958, *T*_max_ = 0.972	*k* = −7→9
8581 measured reflections	*l* = −28→21

### Refinement

**Table:**

Refinement on *F*^2^	Secondary atom site location: difference Fourier map
Least-squares matrix: full	Hydrogen site location: inferred from neighbouring sites
*R*[*F*^2^ > 2σ(*F*^2^)] = 0.042	H-atom parameters constrained
*wR*(*F*^2^) = 0.119	*w* = 1/[σ^2^(*F*_o_^2^) + (0.0641*P*)^2^ + 0.1943*P*] where *P* = (*F*_o_^2^ + 2*F*_c_^2^)/3
*S* = 1.03	(Δ/σ)_max_ < 0.001
2981 reflections	Δρ_max_ = 0.14 e Å^−^^3^
222 parameters	Δρ_min_ = −0.16 e Å^−^^3^
0 restraints	Extinction correction: *SHELXL97* (Sheldrick, 2008), Fc^*^=kFc[1+0.001xFc^2^λ^3^/sin(2θ)]^-1/4^
Primary atom site location: structure-invariant direct methods	Extinction coefficient: 0.0093 (16)

### Special details

**Table:**

Geometry. All e.s.d.'s (except the e.s.d. in the dihedral angle between two l.s. planes) are estimated using the full covariance matrix. The cell e.s.d.'s are taken into account individually in the estimation of e.s.d.'s in distances, angles and torsion angles; correlations between e.s.d.'s in cell parameters are only used when they are defined by crystal symmetry. An approximate (isotropic) treatment of cell e.s.d.'s is used for estimating e.s.d.'s involving l.s. planes.
Refinement. Refinement of *F*^2^ against ALL reflections. The weighted *R*-factor *wR* and goodness of fit *S* are based on *F*^2^, conventional *R*-factors *R* are based on *F*, with *F* set to zero for negative *F*^2^. The threshold expression of *F*^2^ > σ(*F*^2^) is used only for calculating *R*-factors(gt) *etc*. and is not relevant to the choice of reflections for refinement. *R*-factors based on *F*^2^ are statistically about twice as large as those based on *F*, and *R*- factors based on ALL data will be even larger.

### Fractional atomic coordinates and isotropic or equivalent isotropic displacement parameters (Å^2^)

**Table:**

	*x*	*y*	*z*	*U*_iso_*/*U*_eq_	
O1	0.28103 (12)	0.90184 (19)	0.42954 (5)	0.0819 (4)	
O2	0.70723 (11)	0.80481 (15)	0.32588 (5)	0.0638 (3)	
O3	1.12815 (14)	0.4756 (2)	0.43350 (6)	0.0919 (5)	
O4	1.56960 (12)	0.36084 (15)	0.31617 (5)	0.0671 (4)	
O5	1.51234 (12)	0.49882 (14)	0.21947 (5)	0.0609 (3)	
C1	0.2027 (2)	0.8905 (3)	0.48040 (9)	0.0961 (7)	
H1A	0.1911	0.7746	0.4905	0.144*	
H1B	0.1068	0.9414	0.4746	0.144*	
H1C	0.2574	0.9479	0.5104	0.144*	
C2	0.42024 (16)	0.8369 (2)	0.42967 (7)	0.0580 (4)	
C3	0.49109 (19)	0.7598 (3)	0.47532 (7)	0.0698 (5)	
H3	0.4444	0.7487	0.5093	0.084*	
C4	0.63289 (18)	0.6990 (2)	0.46998 (7)	0.0652 (5)	
H4	0.6801	0.6467	0.5011	0.078*	
C5	0.70830 (16)	0.71205 (19)	0.42044 (7)	0.0509 (4)	
C6	0.63254 (15)	0.79194 (18)	0.37421 (6)	0.0482 (4)	
C7	0.49051 (16)	0.8529 (2)	0.37918 (7)	0.0535 (4)	
H7	0.4417	0.9052	0.3484	0.064*	
C8	0.63986 (18)	0.8895 (2)	0.27838 (7)	0.0652 (5)	
H8A	0.5486	0.8345	0.2670	0.098*	
H8B	0.7055	0.8875	0.2476	0.098*	
H8C	0.6199	1.0038	0.2884	0.098*	
C9	0.85599 (16)	0.6388 (2)	0.41978 (7)	0.0556 (4)	
H9	0.8906	0.5935	0.4542	0.067*	
C10	0.94973 (16)	0.6258 (2)	0.37837 (7)	0.0560 (4)	
H10	0.9239	0.6703	0.3429	0.067*	
C11	1.09404 (17)	0.5415 (2)	0.38803 (7)	0.0577 (4)	
C12	1.19894 (16)	0.53612 (18)	0.34148 (6)	0.0490 (4)	
C13	1.17154 (16)	0.61029 (19)	0.28937 (7)	0.0534 (4)	
H13	1.0832	0.6682	0.2822	0.064*	
C14	1.27305 (17)	0.60041 (19)	0.24734 (7)	0.0557 (4)	
H14	1.2519	0.6506	0.2123	0.067*	
C15	1.40530 (16)	0.51638 (18)	0.25735 (7)	0.0504 (4)	
C16	1.43577 (15)	0.44187 (18)	0.31052 (6)	0.0493 (4)	
C17	1.33443 (16)	0.45188 (19)	0.35158 (7)	0.0515 (4)	
H17	1.3555	0.4022	0.3867	0.062*	
C18	1.61263 (19)	0.2977 (2)	0.37027 (8)	0.0669 (5)	
H18A	1.6114	0.3869	0.3976	0.100*	
H18B	1.7109	0.2520	0.3696	0.100*	
H18C	1.5449	0.2111	0.3805	0.100*	
C19	1.4842 (2)	0.5671 (3)	0.16424 (7)	0.0703 (5)	
H19A	1.3940	0.5204	0.1478	0.105*	
H19B	1.5650	0.5401	0.1409	0.105*	
H19C	1.4745	0.6869	0.1668	0.105*	

### Atomic displacement parameters (Å^2^)

**Table:**

	*U*^11^	*U*^22^	*U*^33^	*U*^12^	*U*^13^	*U*^23^
O1	0.0504 (7)	0.1228 (11)	0.0734 (9)	0.0280 (7)	0.0114 (6)	−0.0078 (8)
O2	0.0466 (6)	0.0894 (9)	0.0563 (7)	0.0110 (5)	0.0110 (5)	0.0148 (6)
O3	0.0720 (8)	0.1376 (13)	0.0679 (9)	0.0480 (8)	0.0213 (7)	0.0313 (8)
O4	0.0547 (7)	0.0841 (8)	0.0635 (8)	0.0250 (6)	0.0118 (6)	0.0059 (6)
O5	0.0566 (7)	0.0690 (7)	0.0584 (7)	0.0066 (5)	0.0163 (5)	0.0010 (5)
C1	0.0587 (12)	0.152 (2)	0.0797 (14)	0.0236 (12)	0.0227 (10)	−0.0226 (14)
C2	0.0426 (8)	0.0710 (10)	0.0609 (10)	0.0082 (7)	0.0086 (7)	−0.0105 (8)
C3	0.0573 (10)	0.0980 (14)	0.0555 (10)	0.0150 (9)	0.0167 (8)	0.0014 (9)
C4	0.0584 (10)	0.0837 (12)	0.0541 (10)	0.0146 (9)	0.0096 (8)	0.0066 (9)
C5	0.0459 (8)	0.0538 (9)	0.0534 (9)	0.0033 (7)	0.0081 (7)	−0.0011 (7)
C6	0.0417 (8)	0.0509 (8)	0.0526 (9)	−0.0013 (6)	0.0072 (7)	−0.0032 (7)
C7	0.0446 (8)	0.0592 (9)	0.0564 (9)	0.0043 (7)	0.0001 (7)	−0.0016 (7)
C8	0.0560 (10)	0.0859 (12)	0.0536 (10)	0.0003 (9)	0.0032 (8)	0.0061 (9)
C9	0.0493 (9)	0.0599 (9)	0.0578 (10)	0.0071 (7)	0.0041 (7)	0.0040 (7)
C10	0.0471 (9)	0.0633 (10)	0.0578 (10)	0.0086 (7)	0.0059 (7)	0.0015 (8)
C11	0.0508 (9)	0.0665 (10)	0.0561 (10)	0.0120 (7)	0.0068 (7)	0.0032 (8)
C12	0.0435 (8)	0.0489 (8)	0.0548 (9)	0.0034 (6)	0.0044 (7)	−0.0046 (7)
C13	0.0450 (8)	0.0538 (9)	0.0616 (10)	0.0088 (7)	0.0045 (7)	0.0006 (7)
C14	0.0555 (9)	0.0579 (9)	0.0539 (9)	0.0054 (7)	0.0050 (7)	0.0048 (7)
C15	0.0486 (9)	0.0483 (8)	0.0549 (9)	−0.0010 (6)	0.0103 (7)	−0.0056 (7)
C16	0.0437 (8)	0.0475 (8)	0.0570 (9)	0.0067 (6)	0.0059 (7)	−0.0046 (7)
C17	0.0491 (8)	0.0540 (9)	0.0515 (9)	0.0065 (7)	0.0032 (7)	−0.0009 (7)
C18	0.0581 (10)	0.0706 (11)	0.0717 (12)	0.0165 (8)	0.0009 (8)	0.0029 (9)
C19	0.0722 (11)	0.0831 (12)	0.0566 (10)	0.0015 (10)	0.0141 (8)	0.0019 (9)

### Geometric parameters (Å, °)

**Table:**

O1—C2	1.3593 (19)	C8—H8B	0.9600
O1—C1	1.427 (2)	C8—H8C	0.9600
O2—C6	1.3586 (18)	C9—C10	1.329 (2)
O2—C8	1.420 (2)	C9—H9	0.9300
O3—C11	1.222 (2)	C10—C11	1.473 (2)
O4—C16	1.3707 (18)	C10—H10	0.9300
O4—C18	1.410 (2)	C11—C12	1.487 (2)
O5—C15	1.3567 (17)	C12—C13	1.377 (2)
O5—C19	1.425 (2)	C12—C17	1.405 (2)
C1—H1A	0.9600	C13—C14	1.387 (2)
C1—H1B	0.9600	C13—H13	0.9300
C1—H1C	0.9600	C14—C15	1.379 (2)
C2—C3	1.372 (3)	C14—H14	0.9300
C2—C7	1.385 (2)	C15—C16	1.404 (2)
C3—C4	1.381 (2)	C16—C17	1.369 (2)
C3—H3	0.9300	C17—H17	0.9300
C4—C5	1.387 (2)	C18—H18A	0.9600
C4—H4	0.9300	C18—H18B	0.9600
C5—C6	1.412 (2)	C18—H18C	0.9600
C5—C9	1.456 (2)	C19—H19A	0.9600
C6—C7	1.382 (2)	C19—H19B	0.9600
C7—H7	0.9300	C19—H19C	0.9600
C8—H8A	0.9600		
			
C2—O1—C1	118.09 (15)	C9—C10—C11	120.80 (15)
C6—O2—C8	119.28 (12)	C9—C10—H10	119.6
C16—O4—C18	117.28 (12)	C11—C10—H10	119.6
C15—O5—C19	117.65 (13)	O3—C11—C10	120.98 (14)
O1—C1—H1A	109.5	O3—C11—C12	119.72 (14)
O1—C1—H1B	109.5	C10—C11—C12	119.30 (14)
H1A—C1—H1B	109.5	C13—C12—C17	118.23 (14)
O1—C1—H1C	109.5	C13—C12—C11	123.79 (14)
H1A—C1—H1C	109.5	C17—C12—C11	117.98 (14)
H1B—C1—H1C	109.5	C12—C13—C14	121.35 (14)
O1—C2—C3	124.58 (15)	C12—C13—H13	119.3
O1—C2—C7	115.21 (15)	C14—C13—H13	119.3
C3—C2—C7	120.21 (14)	C15—C14—C13	120.14 (15)
C2—C3—C4	118.93 (16)	C15—C14—H14	119.9
C2—C3—H3	120.5	C13—C14—H14	119.9
C4—C3—H3	120.5	O5—C15—C14	125.17 (15)
C3—C4—C5	123.17 (16)	O5—C15—C16	115.61 (13)
C3—C4—H4	118.4	C14—C15—C16	119.23 (13)
C5—C4—H4	118.4	C17—C16—O4	125.14 (14)
C4—C5—C6	116.59 (14)	C17—C16—C15	120.09 (14)
C4—C5—C9	117.84 (14)	O4—C16—C15	114.77 (12)
C6—C5—C9	125.54 (14)	C16—C17—C12	120.96 (15)
O2—C6—C7	123.12 (14)	C16—C17—H17	119.5
O2—C6—C5	116.28 (13)	C12—C17—H17	119.5
C7—C6—C5	120.60 (14)	O4—C18—H18A	109.5
C6—C7—C2	120.51 (15)	O4—C18—H18B	109.5
C6—C7—H7	119.7	H18A—C18—H18B	109.5
C2—C7—H7	119.7	O4—C18—H18C	109.5
O2—C8—H8A	109.5	H18A—C18—H18C	109.5
O2—C8—H8B	109.5	H18B—C18—H18C	109.5
H8A—C8—H8B	109.5	O5—C19—H19A	109.5
O2—C8—H8C	109.5	O5—C19—H19B	109.5
H8A—C8—H8C	109.5	H19A—C19—H19B	109.5
H8B—C8—H8C	109.5	O5—C19—H19C	109.5
C10—C9—C5	131.00 (16)	H19A—C19—H19C	109.5
C10—C9—H9	114.5	H19B—C19—H19C	109.5
C5—C9—H9	114.5		

### Hydrogen-bond geometry (Å, °)

**Table:**

*D*—H···*A*	*D*—H	H···*A*	*D*···*A*	*D*—H···*A*
C4—H4···O3^i^	0.93	2.46	3.363 (3)	162
C8—H8B···O4^ii^	0.96	2.60	3.537 (3)	166
C10—H10···O2	0.93	2.25	2.846 (3)	121
C19—H19A···O1^iii^	0.96	2.54	3.443 (3)	157

Symmetry codes: (i) −*x*+2, −*y*+1, −*z*+1; (ii) −*x*+5/2, *y*+1/2, −*z*+1/2; (iii) −*x*+3/2, *y*−1/2, −*z*+1/2.
